# Effects of chronic core training on serum and erythrocyte oxidative stress parameters in amputee football players

**DOI:** 10.3389/fphys.2023.1188843

**Published:** 2023-06-09

**Authors:** Ahmet Kurtoğlu, Nurettin Konar, Faruk Akçınar, Bekir Çar, Nuray Üremiş, Yusuf Türköz, Özgür Eken, Halil İbrahim Ceylan, Vera Knappova, Magdalena Barasinska, Tomasz Gabrys

**Affiliations:** ^1^ Department of Coaching Education, Faculty of Sport Science, Bandirma Onyedi Eylul University, Balikesir, Türkiye; ^2^ Department of Physical Education and Sport Teaching, Faculty of Sport Sciences, Bandirma Onyedi Eylul University, Balikesir, Türkiye; ^3^ Department of Coaching Education, Faculty of Sport Science, Inonu University, Malatya, Türkiye; ^4^ Department of Medical Biochemistry, Medical Faculty, Inonu University, Malatya, Türkiye; ^5^ Department of Physical Education and Sport Teaching, Faculty of Sport Sciences, Inonu University, Malatya, Türkiye; ^6^ Department of Physical Education and Sports Teaching, Kazim Karabekir Faculty of Education, Ataturk University, Erzurum, Türkiye; ^7^ Department of Physical Education and Sport, Faculty of Education, University of West Bohemia, Pilsen, Czechia; ^8^ Department of Health Sciences, Jan Dlugosz University, Czestochowa, Poland

**Keywords:** amputee, amputee football, core exercise, total oxidant status, total antioxidant status

## Abstract

**Objective:** The positive impact of aerobic exercise on blood oxidative stress parameters is well documented. However, the effect of core exercises on these parameters in amputee football players (AF) remains unclear. Therefore, this study aims to investigate the impact of core exercises on blood oxidative stress parameters in this population.

**Methods:** Experimental method was adopted in the study. Eleven elite AF players participated in the study. The participants were divided randomly into two groups a core exercise group (CEG) and a control group (CG). Blood measurements were taken before and after the 8-week core exercise program. Blood measurements included erythrocyte Total Oxidant Status (eTOS), erythrocyte Total Antioxidant Status (eTAS), erythrocyte oxidative stress index (eOSI), serum nitric oxide (sNO), serum Total Oxidant Status (sTOS), serum Total Antioxidant Status (sTAS), serum oxidative stress index (sOSI), serum total thiol (sTT), serum native thiol (sNT), and serum disulfide (sDS) parameters were studied.

**Results:** According to the results of the study, a significant difference was found between the 0th and eighth week pre-aerobic training load (ATL) sTOS (*p* = .028) values of CEG values. A significant difference was found in sTOS (*p* = .028) and sOSI (*p* = .028) values after the 0th and eighth-week pre-ATL. A significant difference was found in the sTOS (*p* = .043) and sOSI values (*p* = .043) of CG at week 0th and eighth-week pre-ATL.

**Conclusion:** Overall, the results suggest that core exercises had a positive effect on blood oxidative stress parameters in AF players by reducing blood total oxidant levels.

## 1 Introduction

Under normal physiological conditions, the levels of oxidants and antioxidants are maintained in equilibrium. However, when the balance is disrupted due to an increase in oxidants or a decrease in antioxidants, oxidative stress ensues, leading to cellular damage. Although chronic exercise is known to induce the formation of oxidants and oxidative stress in metabolism, it also triggers the synthesis of antioxidants (Ji, 1995; Suzuki et al., 2020). Investigations examining the association between certain sports and oxidative stress can be traced back to the late 1970s (Radak and Powers, 2020). Studies are showing that oxidative stress also occurs as a consequence of cardiovascular disease (Lorenzon Dos Santos et al., 2020), diabetes (Pitocco et al., 2010), hypertension (Korsager Larsen and Matchkov, 2016), and many neurological diseases (Tönnies and Trushina, 2017). In this context, the impact of exercise on oxidative parameters can be either positive or negative, contingent upon the overall health status of individuals (Magherini et al., 2019).

Oxidative balance is the system of equilibrium between the rate of formation and elimination of free radicals formed as a result of changes in the organism, such as exercise or disease (Fearon and Faux, 2009). Free radicals are reactive atoms or molecules that have one or more unpaired electrons in their outer shell and can be formed by the interaction of oxygen with certain molecules (Chandrasekaran et al., 2017). These molecules have a short lifetime and high reactivity. In this way, the electrons of the free radicals react with the next stable molecule to ensure their stability, resulting in the formation of a new free radical. In this way, other molecules that become unstable cause cellular components to deteriorate due to the action of the free radicals (Martinović et al., 2011). While cellular perturbations cause a decline in muscle function, free radicals that react directly with contractile proteins negatively affect muscle-specific strength formation (Callahan et al., 2001).

Oxygen consumption in skeletal muscle increases 100- to 200-fold during exercise, resulting in increased mitochondrial electron flow (Kawamura et al., 2018). The main source of oxidative stress in athletes is thought to be the increase in the number of oxygen radicals resulting from leakage during electron transfer reactions during exercise (Margaritis et al., 2003). The intensity of exercise is important for the generation of oxidative stress (Alessio et al., 1988; Koz et al., 1992). High-intensity and prolonged exercise increase the amount of free radicals, leading to oxidative muscle damage (Powers et al., 2011). Moderate and regular exercise has been found to cause moderate oxidative stress and have a positive effect on metabolism (Pedersen and Saltin, 2015; Di Meo et al., 2019).

Compared to healthy individuals, the metabolism of amputees is different from healthy individuals (Mao et al., 2022). Studies have reported that individuals with amputation need more energy compared to healthy individuals (Bernardi et al., 1999; Waters and Mulroy, 1999). There is also a positive relationship between the level of amputation and the energy requirement (Gailey et al., 1994). This change in energy needs, oxidant and antioxidant parameters in amputees create different physiological processes in metabolism (Lin et al., 2010). Peivandi Yazdi et al. (2018) reported a decrease in serum TAS level after amputation surgery. In conjunction with increased energy requirements, this decrease in TAS levels may negatively impact daily functional mobility. Therefore, it is believed that chronic exercise may be an important way to prevent oxidative damage in amputees. However, considering the vascular and metabolic differences in amputees, it is controversial which type of exercise has a positive effect on the oxidative balance. Therefore, the aim of this study is to investigate the effects of an 8-week core exercise program on oxidative and antioxidant changes in amputees. In this context, the research hypothesis was set as “Chronic core exercise reduces erythrocyte and serum levels TOS”.

## 2 Materials and methods

### 2.1 Participants

The present study followed a within-subjects design, in which two or more measurements were collected from a sample of subjects and groups. The minimum sample size was calculated using G-power software 3.1.9.7 (University of Dusseldorf, Dusseldorf, Germany) (Kang, 2021). According to this analysis, t tests [Means: Difference between two dependent means (matched pairs)] were used to calculate power following our study design; within-factors; *α* err prob = 0.05; minimum effect size = 1.8, and power (1-β err prob) = 0.80. Accordingly, when the actual power was taken as 80.7%, it was determined that there should be at least five people for each group. Therefore, a total of 11 athletes participated in this study voluntarily. Participants were randomly divided into two groups as core exercise group (CEG, *n* = 6) and control group (CG, *n* = 5). Baseline characteristic of the study population is given in Table 1. CEG had participants with two transtibial, two knee disarticulation, one arm amputation, and one hip disarticulation. There were three transtibial, one knee disarticulation, one arm amputation, and one hip disarticulation participants in CG. In the study, the experimental method, in which the participants were randomly selected from the quantitative data collection techniques, was used (Keppel, 1991). The necessary permissions for the study were obtained from the management of the Malatya Amputee Football Team. The study was conducted in accordance with the criteria of the Declaration of Helsinki. The study was approved by the Malatya Clinical Research Ethics Committee by Decision No. 2020/142. Participants were informed about the training programme and tests to be performed as part of the study. Participants were informed that they could withdraw from the study at any time. In this context, Malatya Amputee Football Team (*n* = 14), one of the amputee football super league teams, was included in the research in the 2020-2021 season. The study participants were 1) with cardiovascular disease 2) with a chronic respiratory problem 3) with the consumption of harmful substances such as cigarettes, and alcohol 4) with hypertension 5) with coronary artery disease 6) with a diagnosis of cardiac arrhythmia 7) with active infection not included. In this context, three participants with diagnoses a (*n* = 1), c (*n* = 1), and g (*n* = 1) were not included in the study.

**TABLE 1:** Baseline characteristics of the study population.

Parameters	CEG (*n* = 6) x– ±SD	CG (*n* = 5) x– ±SD	*p*
Age (year)	23.80 ± 4.20	28.33 ± 6.63	*p* > 0.05
Height (cm)	175.16 ± 9.66	174.40 ± 11.54	*p* > 0.05
Weight (kg)	72.60 ± 15.85	61.40 ± 3.91	*p* > 0.05
Body mass index (kg/m^2^)	23.51 ± 3.76	20.38 ± 3.23	*p* > 0.05

### 2.2 Design and procedures

**FIGURE 1:**
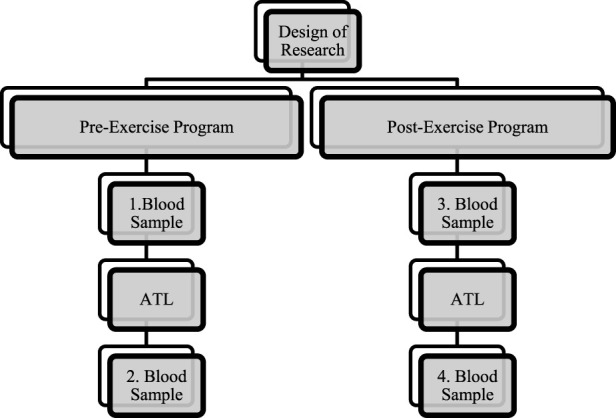
Experimental design.

Before the start of the study, initial blood measurements were taken in 0th week before aerobic training load (ATL). After 30 min, 60 min of ATL (10 min of warm-up, 5 min of stretching, 15 min of aerobic jogging (heart rate (HR) < 60%), 5 min of rest, 15 min of aerobic jogging (HR < 60%), 5 min of jogging, 5 min of stretching) was performed (Karvonen and Vuorimaa, 1988). The second blood measurement was performed 30 min after ATL. After the first blood measurements, an 8-week core training program was performed. At the end of the eighth week, the blood measurements were repeated in the same order (Figure 1).

#### 2.2.1 Data collection

Participants’ blood samples were collected by specialized nurses in a private hospital laboratory. Blood samples were collected from the superficial veins of the arm (median cubital, basilic, and cephalic veins) using a sterile injector and blood collection method via a vacuum tube and needle system. The collected blood samples were placed in yellow 4-mm EDTA tubes with lids. The blood in the tubes was centrifuged using a Hettich Rotofiz 32 A centrifuge at 2000 RPM at +4°C for 10 min. After centrifugation, the plasma and blood cell pack fractions were collected and filled into eppendorfs sample tubes. Then, erythrocytes were washed with 0.9% NaCl solution three times, and washed erythrocytes were haemolysed by dilution with deionized water (50-fold). The plasma and erythrocytes samples were also divided into ependorfs sample tubes and stored at −80 degrees until analysis. After the last blood draw, eTOS, eTAS, eOSI, sNO, sTOS, sTAS, sOSI, sTT, sNT, and sDS levels of the plasma and erythrocytes samples were analyzed using commercial kits (24). Using a spectrophotometer (microplate reader; Synergy H1) and commercially available kits, the Total Antioxidant Status (TAS), Total Oxidant Status (TOS), Native Thiol (NT), and Total Thiol (TT) levels in the samples were measured (Rel Assay Diagnostics, Gaziantep, Turkey). The ratio of TOS to TAS was accepted as OSI. The OSI value was calculated according to the following formula: OSI (arbitrary unit) = TOS (micromole H2O2 Eqv/L)/TAS (millimole Trolox Eqv/L) (Erel, 2005). The NO level of the supernatant was measured as total nitrite by Griess reaction. Measurements were made according to the method previously adopted byOzbek et al. (2000).

#### 2.2.2 BMI Measurements of Participants

BMI calculation of disabled population differs from nondisabled population. For this reason, BMIs of amputees were corrected according to the Amputee Coalition (AC). Based on the published sources and expert opinion, AC calculator corrects for proportions of total BM missing based on the following percentages: foot (Symes) = 1.30%, transtibial = 3.26%, transfemoral = 9.96%, and hip disarticulation/hemipelvectomy = 11.83%. This correction does not differ for men or women, unlike other estimates which are slightly higher for women: 3.355% and 10.712% (transtibial and transfemoral, respectively). Estimated BM was calculated as; Estimated BM= (BM without prosthesis)/(1.0−AC% converted to decimal fraction) (Osterkamp, 1995; Durkin and Dowling, 2003; Frost et al., 2017).

#### 2.2.3 Core exercise programme

The core training program (Table 2) was performed on participants concurrently with their regular season training regimen as amputee football players. Following a 10-min warm-up and 5-min stretching session, CEG participants performed between four to five core exercises. After core training, participants continued their normal football training. For 8 weeks, CEG and CG did the training warm-up and stretching exercises together. He was then taken to CEG core training, while CG continued to warm up with the soccer ball.

**TABLE 2:** Core exercise program.

Weeks	1 Day	2 Day	3 Day
1 Week	Bicycle crunch (10 × 3 sets)	Prone plank (3 × 15 s)	Russian twist (10 × 3 sets)
	Reverse crunch (10 × 3 sets)	Stability ball plank (3 × 15 s)	Flutter kick (10 × 3 sets)
	Bird dog (10 × 3 sets)	Right side bridge (3 × 15 s)	Side double-leg lift (10 × 3 sets)
	Reverse pendelum (10 × 3 sets)	Left side bridge (3 × 15 s)	Swimmer (10 × 3 sets)
		Back plank (3 × 15 s)	
2 Week	Superman (15 × 3 sets)	Ball rotating crunch (3 × 20 s)	Sıt-Up (15 × 3 sets)
	Leg lower (15 × 3 sets)	Ball supine bridge (3 × 20 s)	Rigt side lateral raise (15 × 3 sets)
	Side to side twist (15 × 3 sets)	Ball hyperextension (3 × 20 s)	Left side lateral raise (15 × 3 sets)
	Dumbel side bend (15 × 3 sets)	Ball crunch (3 × 20 s)	Press-up (15 × 3 sets)
3 Week	Open-book rıb cage (15 × 3 sets)	Superman with medicine Ball (3 × 20 s)	Plate V-Up (15 × 3 sets)
	Hanging knee raise (15 × 3 sets)	Diagonal medicine ball chop (15 × 3 sets)	Jackknife (15 × 3 sets)
	Three way hanging knee raise (15 × 3 sets)	Medicine ball pullover pass (15 × 3 sets)	Sit-Up (15 × 3 sets)
	Static back extension (15 × 3 sets)	Medicine ball overhead throw (15 × 3 sets)	Back extension (15 × 3 sets)
4 Week	Assisted squat (10 × 3 sets)	Waiting back extension (3 × 30 s)	Vertical leg crunch (20 × 3 sets)
	Side to side twist (20 × 3 sets)	Waiting toe tabs (3 × 30 s)	Vertical leg rotation (20 × 3 sets)
	Push-Up (10 × 3 sets)	Waiting leg lower (3 × 30 s)	Dumbell side bend (20 × 3 sets)
	Slide-board thrust (15 × 3 sets)	Waiting leg lower with seated rotation (3 × 30 s)	Glute ham raise (20 × 3 sets)
5 Week	Bicycle crunch (20 × 3 sets)	Prone plank (3 × 30 s)	Russian twist (20 × 3 sets)
	Reverse crunch (20 × 3 sets)	Stability ball plank (3 × 30 s)	Flutter kick (20 × 3 sets)
	Bird dog (20 × 3 sets)	Right side bridge (3 × 30 s)	Side double-leg lift (20 × 3 sets)
	Reverse pendelum (20 × 3 sets)	Left side bridge (3 × 30 s)	Swimmer (20 × 3 sets)
		Back plank (3 × 30 s)	
6 Week	Superman (20 × 3 sets)	Ball rotating crunch (3 × 30 s)	Sıt-Up (20 × 3 sets)
	Leg lower (20 × 3 sets)	Ball supine bridge (3 × 30 s)	Rigt side lateral raise (20 × 3 sets)
	Side to side twist (20 × 3 sets)	Ball hyperextension (3 × 30 s)	Left side lateral raise (20 × 3 sets)
	Dumbel side bend (20 × 3 sets)	Ball crunch (3 × 30 s)	Press-up (20 × 3 sets)
		Ball Plank (3 × 30 s)	
7 Week	Open-book rıb cage (20 × 3 sets)	Superman with medicine ball (3 × 30 s)	Plate V-Up (20 × 3 sets)
	Hanging knee raise (20 × 3 sets)	Diagonal medicine ball chop (20 × 3 sets)	Jackknife (20 × 3 sets)
	Three way hanging knee raise (20 × 3 sets)	Medicine ball pullover pass (20 × 3 sets)	Sit-Up (20 × 3 sets)
	Static back extension (20 × 3 sets)	Medicine ball overhead throw (20 × 3 sets)	Back extension (20 × 3 sets)
8 Week	Assisted squat (15 × 3 sets)	Waiting back extension (3 × 40 s)	Vertical leg crunch (25 × 3 sets)
	Side to side twist (25 × 3 sets)	Waiting toe tabs (3 × 40 s)	Vertical leg rotation (25 × 3 sets)
	Push-Up (15 × 3 sets)	Waiting leg lower (3 × 40 s)	Dumbell side bend (25 × 3 sets)
	Slide-board thrust (20 × 3 sets)	Waiting leg lower with seated rotation (3 × 40 s)	Glute ham raise (25 × 3 sets)

### 2.3 Statistical analysis

SPSS package program 25 was used for data analysis in the study. During normality analysis, it was found that the Skewnes-Kurtosis values were not between (−1.5-+1.5) and the data were not normally distributed (Mardia, 1974). For this reason, the Wilcoxon test, one of the nonparametric tests, was used for statistical analysis. The significance level in the study was set at 0.05.

## 3 Results

Upon analyzing Table 3, a statistically significant difference was observed between the sTOS value of CEG participants during the 0th and eighth weeks pre-ATL (*p = .028*). Additionally, a significant difference was identified in the sTOS (*p = .028*) and sOSI values (*p = .028*) of CEG participants during the 0th and eighth weeks post-ATL.

**TABLE 3:** Comparison of oxidative stress parameters of CEG.

Parameters	Week	Pre-ATL	ES	*p*	Post-ATL	ES	P
eTOS (μmol H_2_O_2_ Equiv/g hemoglobin)	0 Week	323.45 ± 18.69	1.572	0.116	312.11 ± 8.29	1.153	0.249
	8 Week	311.75 ± 14.95			322.56 ± 20.23		
eTAS (mmol Trolox Equiv./g hemoglobin)	0 Week	0.22 ± 0.016	0.210	0.833	0.23 ± 0.013	0.314	0.753
	8 Week	0.22 ± 0.011			0.23 ± 0.018		
eOSI (mmol Trolox/mmol H_2_O_2_)	0 Week	1,445.47 ± 183.26	0.943	0.345	1,332.35 ± 118.91	0.524	0.6
	8 Week	1,381.17 ± 126.70			1,408.15 ± 196.94		
sNO (μmol/L)	0 Week	148.12 ± 1.45	0.316	0.752	149.79 ± 3.69	1.153	0.249
	8 Week	147.94 ± 1.84			148.24 ± 1.95		
sTOS (μmol H_2_O_2_ Equiv/L)	0 Week	4.58 ± 0.72	2.201	.028*	5.73 ± 1.14	2.201	.028*
	8 Week	3.65 ± 0.50			3.85 ± 0.65		
sTAS (mmol Trolox Equiv./L)	0 Week	1.88 ± 0.15	1.572	0.116	1.91 ± 0.16	0.631	0.528
	8 Week	1.69 ± 0.19			1.86 ± 0.20		
sOSI (mmol Trolox/mmol H_2_O_2_)	0 Week	2.43 ± 0.40	1.363	0.173	2.98 ± 0.43	2.201	.028*
	8 Week	2.16 ± 0.13			2.11 ± 0.52		
sTT (µmol/L)	0 Week	318.52 ± 41.52	1.483	0.138	341.48 ± 39.31	0.42	0.674
	8 Week	349.14 ± 19.40			350.90 ± 32.11		
sNT (µmol/L)	0 Week	242.82 ± 24.49	0.736	0.462	271.28 ± 28.00	1.483	0.138
	8 Week	265.58 ± 37.48			292.08 ± 35.02		
sDS (µmol/L)	0 Week	37.85 ± 19.39	0.524	0.6	35.09 ± 23.06	0.135	0.893
	8 Week	41.77 ± 14.33			29.40 ± 8.48		

eTOS: Erythrocyte Total Oxidant Status, eTAS: Erythrocyte Total Antioxidant Status, eOSI: Erythrocyte Oxidative Stress Index, sNO: Serum Nitric Oxid, sTOS: Serum Total Oxidant Status, sTAS: Serum Total Antioxidant Status, sOSI: Erythrocyte Oxidative Stress Index, sTT: Serum Total Thiol, sNT: Serum Native Thiol, sDS: Serum Disulphide, ATL: Aerobic Training Load

Upon analyzing Table 4, a statistically significant difference was observed in the sTOS (*Z = -2.023, p = 0.043*) and sOSI (*Z = -2.023, p = 0.043*) values of the CG participants prior to the 0th and eighth week of pre-ATL. However, no significant difference was found in oxidative stress values following the 0th and eighth weeks of post-ATL in the CG (*p* > 0.05).

**TABLE 4:** Comparison of oxidative stress parameters of CG.

Parameters	Week	Pre-ATL	Z	*p*	Post-ATL	Z	*p*
eTOS (μmol H_2_O_2_ Equiv/g hemoglobin)	0 Week	325.73 ± 33.58	1.21	0.225	299.07 ± 15.58	0.41	0.686
	8 Week	315.25 ± 22.72			320.65 ± 43.39		
eTAS (mmol Trolox Equiv./g hemoglobin)	0 Week	0.23 ± 0.027	1.08	0.279	0.23 ± 0.007	0.27	0.786
	8 Week	0.22 ± 0.015			0.23 ± 0.033		
eOSI (mmol Trolox/mmol H_2_O_2_)	0 Week	1,417.92 ± 234.57	0.67	0.5	1,258.50 ± 90.67	0.67	0.5
	8 Week	1,397.88 ± 180.34			1,438.85 ± 412.19		
sNO (μmol/L)	0 Week	148.70 ± 0.56	1.84	0.066	150.63 ± 5.16	0.68	0.498
	8 Week	146.03 ± 2.60			149.96 ± 1.23		
sTOS (μmol H_2_O_2_ Equiv/L)	0 Week	4.88 ± 1.23	2.02	.043*	5.26 ± 2.39	0.94	0.345
	8 Week	3.39 ± 0.52			3.89 ± 0.89		
sTAS (mmol Trolox Equiv./L)	0 Week	1.85 ± 0.13	1.83	0.068	1.96 ± 0.24	1.75	0.08
	8 Week	1.67 ± 0.28			1.83 ± 0.34		
sOSI (mmol Trolox/mmol H_2_O_2_)	0 Week	2.67 ± 0.80	2.02	.043*	2.67 ± 1.10	0.94	0.345
	8 Week	2.06 ± 0.39			2.14 ± 0.40		
sTT (µmol/L)	0 Week	311.57 ± 15.07	1.48	0.138	335.59 ± 17.66	0.41	0.686
	8 Week	364.56 ± 95.22			339.83 ± 37.99		
sNT (µmol/L)	0 Week	210.86 ± 9.81	1.83	0.068	248.04 ± 21.40	1.21	0.225
	8 Week	266.41 ± 62.03			276.42 ± 18.10		
sDS (µmol/L)	0 Week	50.35 ± 8.65	0.14	0.893	43.77 ± 11.67	1.63	0.104
	8 Week	49.07 ± 23.83			31.70 ± 12.65		

eTOS: erythrocyte total oxidant status, eTAS: erythrocyte total antioxidant status, eOSI: erythrocyte oxidative stress index, sNO: serum nitric oxid, sTOS: serum total oxidant status, sTAS: serum total antioxidant status, sOSI: erythrocyte oxidative stress index, sTT: serum total thiol, sNT: serum native thiol, sDS: serum disulphide, ATL: aerobic training load.

**FIGURE 2:**
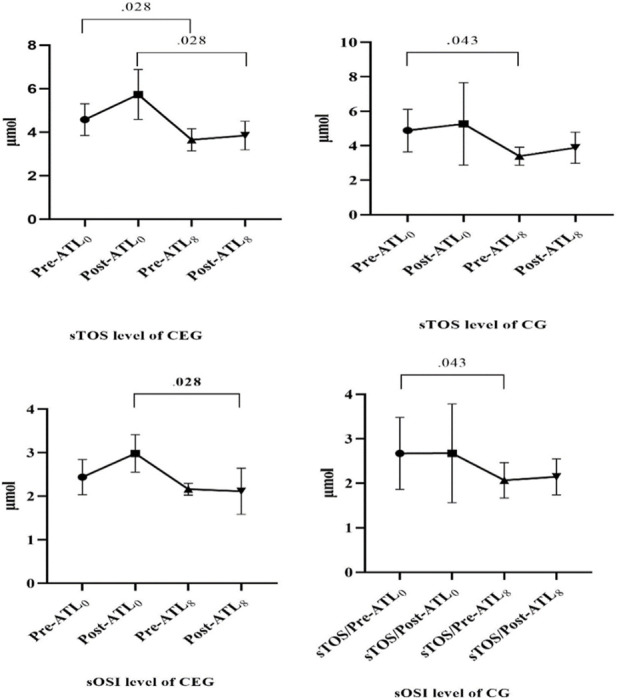
sTOS and sOSI levels of groups.

Figure 2 shows the sTOS values of the groups. As a result of the analysis, a significant difference was found between sTOS-1 and sTOS-3 of CEG and CG. However, the significance level was higher in favor of CEG *(p = 0.028).* When the relationship between sTOS-2 and sTOS-4 was examined, a significant difference was found in favor of CEG *(p = 0.028).* As a result of the analysis, a significant difference was found between sOSI-2 and sOSI-4 in favor of CEG *(p = 0.028).* A significant difference was found between sOSI-1 and sOSI-2 in CG *(p = 0.043).*

## 4 Discussion

In our study, sTOS and sOSI, which are among the oxidative stress parameters in CEG, showed a significant decrease after the 8-week core training program. Although there was a significant difference in CG, the significance level was higher in CEG. As far as we know, this study is the first for amputee football players. Therefore, our hypothesis “core training has a positive effect on oxidative parameters in amputee football players” was confirmed in our study.

It is well known that endurance exercise and high-intensity training activate reactive oxygen species (ROS), which leads to oxidative damage (Radak and Powers, 2020). In other words, oxidative stress is the result of an imbalance between the formation of ROS species and their removal by antioxidant mechanisms (Arazi et al., 2021). It is known that many factors (nutritional status, disease, current training status) play a role in the development of oxidative stress. Therefore, it is unclear which type of exercise increases oxidative stress more. In the study by Hadžović-Džuvo et al. (2014), it was found that there was no difference between the parameters of oxidative stress of 39 top athletes from different sports branches (basketball, football, wrestling). As a result of this study, it was found that the type of sport branches has no influence on the parameters of oxidative stress.

Some studies have looked at the acute and chronic effects of oxidative stress after exercise. Bloomer and Goldfarb examined the effects of anaerobic exercise on acute effects; they concluded that isometric exercises do not cause oxidative stress, that ROS increases as a result of eccentric and isotonic exercise and sprint exercise, and accordingly, an increase in oxidative parameters occurs (Bloomer and Goldfarb, 2004). In the study by Martinović et al. (2011), it was found that oxidative stress parameters increased in elite volleyball players as a result of the 6-week training program. It has long been known that acute endurance exercise increases acute oxidative stress parameters (Alessio et al., 1988). Kawamura et al. (2018) found that low, high, and increasing-intensity endurance running increased acute TAS in animal studies. In Prasertsri and Boonla’s study examining 8-week resistance exercise and high-intensity interval training, the effect of oxidative stress parameters was examined; both training programs were found to increase athletic performance equally, but resistance exercise was found to increase oxidative stress parameters more (Prasertsri and Boonla, 2021). Juergenson et al. examined the effect of 12 weeks of resistance training on oxidative stress parameters and aortic stiffness, it was found that sTAS decreased and sOSI increased after training. This study also found that oxidative stress parameters were largely associated with aortic stiffness (Jürgenson et al., 2019). In the study by Park and Kwak, which examined the effects of aerobic and anaerobic exercise on oxidative parameters, it was found that sTAS did not differ between the two exercise groups and the control group (Park and Kwak, 2016). When these results were evaluated, it was found that aerobic exercise decreased oxidative stress parameters. According to the results of the literature, moderate and light exercises performed at an HRmax of 70% have a positive effect on oxidative parameters (Ito et al., 2013; Hasegawa et al., 2018; Kayacan et al., 2021). When the literature is examined, it has been seen that acute exercises performed with intense intensity increase oxidative stress. However, it has been reported that oxidative stress occurs less in chronic exercises with the strengthening of the metabolism and the increase in the level of physical fitness (Akman et al., 2013). When examined in this context, the results of our study support the literature and after 8 weeks of exercise, the level of sTOS in CEG increased less after loading and the level of sTAS’ increased.

Based on the above-mentioned studies, there have been conflicting results regarding the effect of regular exercise on oxidative parameters. However, in the current study, a decrease in oxidative stress markers was observed following regular resistance exercise. In line with our findings, the previous study reported that long-term regular resistance exercises, similar to aerobic exercises, increased the levels of antioxidant enzymes and decreased lipid peroxidation and oxidative stress levels (Çakir Atabek, 2011). Likewise, a recent systematic review also reported an improvement in oxidative stress parameters following regular resistance exercise. It was stated that this reduction is largely due to the chronic exercise stimulating the increase plasma antioxidants and subsequent reactive oxygen species (ROS) scavenging activities. Moreover, the study further notified that the type of exercise is important for the oxidative stress induced by exercise, along with the total exercise volume (Thirupathi et al., 2021). Additionally, Kalvandi et al. (2022) demonstrated that the total antioxidant capacity, superoxide dismutase, and glutathione peroxidase markers developed after resistance exercises (8-week elastic resistance training (three times per week on non-consecutive days for 8 weeks) led to a decrease in the concentration of Malondialdehyde (the lipid peroxidation index). The same researchers proposed that increased adaptations related to lipid peroxidation after resistance exercises trigger the decrease in oxidative stress parameters (Kalvandi et al., 2022). Lastly, in literature, various mechanisms have been proposed to explain the decrease in oxidative stress parameters after regular resistance exercise. For instance; in previous studies, it was proposed that the reduction in oxidative stress parameters following regular resistance exercise may be associated with exercise-induced redox-linked health adaptations through the upregulation of the antioxidant defense system (Radak et al., 2013; de Lima et al., 2021). In a review study, the mechanism behind the improvement in antioxidant capacity and the resulting decrease in oxidative stress levels after resistance exercise was explained as follows; after resistance exercise, both metabolic-mechanical stress and ROS production are induced. These stimuli primarily activate different signaling pathways, mainly within the skeletal muscle, leading to the activation of transcription factors that initiate the transcription and translation of antioxidant proteins. Therefore, when a person exercises periodically, the transcription and translation of these antioxidants become cumulative, resulting in an increase in both muscle and systemic antioxidant capacity and a decrease in basal oxidative stress levels (Gacitua et al., 2018).

NO plays an important role in human metabolism. NO provides vasodilation of blood vessels, increases blood flow, controls mitochondrial respiration, and thus increases performance. Therefore, there is a positive relationship between exercise and NO (Moncada and Higgs, 2006). Qi et al. and Ito et al. found that 4 weeks of gradual jogging at a HR_max_ of 70% increased the level of NO in rats (Ito et al., 2013; Qi et al., 2020). Hasegawa et al. concluded that high-intensity interval training (HITT) increased NO levels more than aerobic exercise and strength training (Hasegawa et al., 2018). It is suggested that the reason for the different results compared to the literature is the training method (core training) and type (aerobic training) chosen.

Kayacan et al. (2021) investigated the effects of high, moderate, and low-intensity exercise on the oxidative parameters sTT, sNT, and sDS. The results of the study showed that sTT, sNT, and sDS levels were lower in moderate and light exercise than in the control and HITT groups. Although sTT, sNT, and sDS differed on average, this difference was not considered significant (*p* > 0.05). In this context, the results of our study and the results of the study conducted by Kayacan show similarities.

According to the research results, core training has a positive effect on the oxidative parameters of sTOS and sOSI in amputee football players. It was found that there were differences in the mean value of some parameters, but no significant differences. In this regard, it is believed that different results may be obtained in studies with larger sample groups. This research was conducted on individuals with different amputation rates. It is thought that studies to be conducted according to the amputation rate in larger participant groups will contribute to the literature. Another limitation of the study is that sedentary amputees were not included in the study. It can be investigated how the physiological process in sedentary amputees will change compared to athlete amputees.

## 5 Conclusion

It is postulated that the positive impact of core training on oxidative parameters in amputee football players can be attributed to physiological alterations in the cardiovascular system that arise from amputation. Consequently, it is recommended to explore the potential correlation between cardiac biomarkers in amputees and healthy subjects. In addition, it is thought that studies comparing different exercise types will contribute to the literature. Studies to be carried out considering the level of amputation will contribute to the selection of the right exercise for amputees. Simim et al., 2013

## Data Availability

The original contributions presented in the study are included in the article/Supplementary Material, further inquiries can be directed to the corresponding authors.
